# A serum panel of three microRNAs may serve as possible biomarkers for kidney renal clear cell carcinoma

**DOI:** 10.1186/s12935-023-03187-z

**Published:** 2024-01-08

**Authors:** Zhenyu Wen, Yingqi Li, Zhengping Zhao, Rongkang Li, Xinji Li, Chong Lu, Chen Sun, Wenkang Chen, Zhenjian Ge, Liangchao Ni, Yongqing Lai

**Affiliations:** 1 Guangdong and Shenzhen Key Laboratory of Reproductive Medicine and Genetics, Department of Urology, Peking University Shenzhen Hospital, 1120 Lianhua Road, Shenzhen, 518036 Guangdong People’s Republic of China; 2 Shantou University Medical College, Shantou, 515063 Guangdong China; 3 Shenzhen University, Shenzhen, 518055 Guangdong China; 4 Anhui Medical University, Hefei, 230032 Anhui China

**Keywords:** MicroRNA, Kidney renal clear cell carcinoma, Diagnosis, Biomarker, Bioinformatics

## Abstract

**Background:**

Although non-invasive radiological techniques are widely applied in kidney renal clear cell carcinoma (KIRC) diagnosis, more than 50% of KIRCs are detected incidentally during the diagnostic procedures to identify renal cell carcinoma (RCC). Thus, sensitive and accurate KIRC diagnostic methods are required. Therefore, in this study, we aimed to identify KIRC-associated microRNAs (miRNAs).

**Methods:**

This three-phase study included 224 participants (112 each of patients with KIRC and healthy controls (NCs)). RT-qPCR was used to evaluate miRNA expression in KIRC and NC samples. Receiver operating characteristic (ROC) curves and the area under the ROC curve (AUC) were used to predict the usefulness of serum miRNAs in KIRC diagnosis. In addition, we performed survival and bioinformatics analyses.

**Results:**

We found that miR-1-3p, miR-129-5p, miR-146b-5p, miR-187-3p, and miR-200a-3p were significantly differentially expressed in patients with KIRC. A panel consisting of three miRNAs (miR-1-3p, miR-129-5p, and miR-146b-5p) had an AUC of 0.895, ranging from 0.848 to 0.942. In addition, using the GEPIA database, we found that the miRNAs were associated with *CREB5*. According to the survival analysis, miR-146b-5p overexpression was indicative of a poorer prognosis in patients with KIRC.

**Conclusions:**

The identified three-miRNA panel could serve as a non-invasive indicator for KIRC and *CREB5* as a potential target gene for KIRC treatment.

## Background

Renal cell carcinoma (RCC) accounts for approximately 2% of all cancer cases worldwide. The incidence of RCC in high/very high Human Development Index (HDI) countries is higher than that in low/medium HDI Countries [1]. In America, over the past few decades, the prevalence of RCC has doubled [2]. Kidney renal clear cell carcinoma (KIRC) is the most common pathological subtype, accounting for approximately 75% of all RCC cases [3]. With the use of minimally invasive image-guided radiological techniques, early and small RCCs, including a large majority of KIRC cases, can be diagnosed. Examples of the techniques include ultrasonography (US) and computed tomography (CT) [4]. Because of the classic triad of flank discomfort, gross hematuria, and palpable abdominal inconspicuousness, approximately 50% of KIRCs are incidentally discovered using frequent non-invasive radiographic techniques [4]. In addition, the utilization of non-invasive radiological techniques to diagnose early and tiny KIRC necessitates the significant experience of the attending physician and patients with good renal function [5]. Therefore, more sensitive and accurate KIRC detection methods are required. microRNAs, also known as miRNAs, are endogenous non-coding RNAs that regulate gene expression. miRNAs are abundant in a variety of body fluids such as serum and urine [6, 7]. miRNAs are involved in the development and progression of KIRC [8–10]. Consequently, miRNAs can be used as serum biomarkers for tumor diagnosis [11]. In this study, we aimed to identify miRNA biomarkers for KIRC diagnosis and prognosis.

## Methods

### Study participants and sample collection

A total of 224 volunteers that visited the Peking University Shenzhen Hospital, including 112 patients with KIRC and 112 healthy controls (NCs) were enrolled in the study (Table 1). All patients with KIRC were histologically diagnosed and untreated. The healthy controls were free of chronic diseases, including cancer. Peripheral blood (5–10 mL) was collected from each participant. Serum was isolated from the obtained blood samples and maintained at −80 °C until further use.

**Table 1:** Characteristics of the study population

	Training phase (n = 56)			Validation phase (n = 168)		
	KIRC	NC		KIRC	NC	
Total number	28	28		84	84	
Age at diagnosis			p = 0.68			p = 0.38
	58.93 ± 14.0	60.4 ± 13.0		62.19 ± 12.7	60.4 ± 13.5	
Gender			p = 0.79			p = 0.21
Male	13 (46.4%)	12(42.9%)		48 (57.1%)	41 (48.9%)	
Female	15 (53.6%)	16(57.1%)		36 (42.9%)	43 (51.1%)	
Location						
Left	16 (57.1%)			43 (51.1%)		
Right	12 (42.9%)			41 (48.9%)		
Fuhrman grade						
Grade I	4 (14.3%)			10 (11.9%)		
Grade II	14 (50.0%)			50 (59.5%)		
Grade III	8 (28.6%)			20 (23.8%)		
Grade IV	2 (7.1%)			4 (4.8%)		
AJCC clinical stage						
Stage I	17 (60.7%)			68 (80.9%)		
Stage II	8 (28.6%)			10 (11.9%)		
Stage III	1 (3.6%)			4 (4.8%)		
Stage IV	2 (7.1%)			2 (2.4%)		

### Study design

The study design was approved by the ethics review board of the Peking University Shenzhen Hospital.

The study was divided into three phases: candidate biomarker identification, screening of identified biomarkers in a sub-group of the study participants, and determining the efficacy of the miRNAs in KIRC diagnosis.

Phase 1: The Gene Expression Omnibus and PubMed databases were searched for miRNAs that were significantly expressed in KIRC and selected as candidate miRNAs. Subsequently, potential miRNAs were selected using the Encyclopedia of RNA Interactomes (ENCORI) database. The criteria were as follows: p less than 0.05 and a fold change (FC) of either greater than 1 or less than -1 [12].

Phase 2: During this phase (Training Phase), the expression of the miRNAs identified in phase 1 were compared between serum samples from NCs and KIRCs (n = 28 each) using RT-qPCR and the 2 − △△Cq method.

Phase 3: During this phase (Validation Phase), the identified miRNA biomarkers were used to confirm KIRC diagnosis efficacy in KIRC and NC serum samples (n = 84 each) using RT-qPCR and the 2 − △△Cq method. To confirm the diversity of miRNAs between KIRCs and NCs, receiver operating characteristic curve (ROC) analysis was performed and the diagnostic ability of miRNAs were determined using area under the curve (AUC) values. Finally, a backward stepwise logistic regression method was used to create an ideal model to obtain the final candidate biomarkers.

### RNA extraction, cDNA synthesis, and RT-qPCR

Synthetic *Caenorhabditis elegans*-derived miR-54 (cel-miR-54-5p) (2 µL, 10 nM, RiboBio, China) was added to each serum sample. Total RNA was extracted from the serum using the TRIzol LS isolation kit (Invitrogen, USA), according to the manufacturer’s instructions. Subsequently, total RNA was lysed with 30 µl RNase-free water and stored at − 80 °C. The concentration miRNA was measured using a NanoDrop 2000c (Thermofisher, USA). Next, the miRNAs were amplified using reverse transcription-specific primers (HaiGene, China) from the bulge-loop miRNA RT-qPCR Primer Set. RT-qPCR was performed using the LightCycler 480 Real-Time PCR System (Roche Diagnostics, Germany) with the TaqMan probe (Sangon, China). Relative target miRNA expression was determined using the 2 − △△Cq method [13]. miRNA expression levels were normalized to those of spiked-in cel-miR-54, as previously described [14]. The miRNAs with Cq values less than 35 were included in the data analysis.

### Bioinformatic analysis

We used the MiRWalk3.0 database (http://mirwalk.umm.uni-heidelberg.de/) to select target genes that were related to two or more of the most common candidate miRNAs [15]. Subsequently, enrichment analysis of the target genes related to two or more candidate miRNAs was performed using the Enrichr database for gene ontology (GO) functional annotation and Kyoto Encyclopedia of Genes and Genomes (KEGG) pathway analyses [16]. Next, we investigated the target genes related to all candidate miRNAs in KIRC using the GEPIA database. The screening criteria were as follows: matched TCGA normal and GTEx data, and |log2FC|> 1, p < 0.01 [17]. Lastly, we performed Kaplan–Meier survival analysis and log-rank test on patients in the OncoLnc database to predict the overall survival rate of patients with KIRC [18].

### Statistical analysis

We analyzed the data using SPSS 20.0. Student’s t-test and a Mann–Whitney test were used for KIRC and NC comparison. In addition, multivariate logistic regression analysis was used to construct an miRNA signature panel. ROC curves and AUC, generated by multiple logistic regression analysis were used to evaluate the diagnostic capabilities of serum miRNAs. AUC values ranging from 0.5 to 0.7 were considered low, 0.7 to 0.85 moderate, and 0.85 to 1.0 high.

## Results

### Population characteristics

In terms of age and sex, there were no significant differences between the healthy controls and patients with KIRC (Table 1). There were no significant age or sex differences in the KIRC and NC groups in phase 2 and 3.

### Identification of candidate miRNAs

**Fig. 1:**
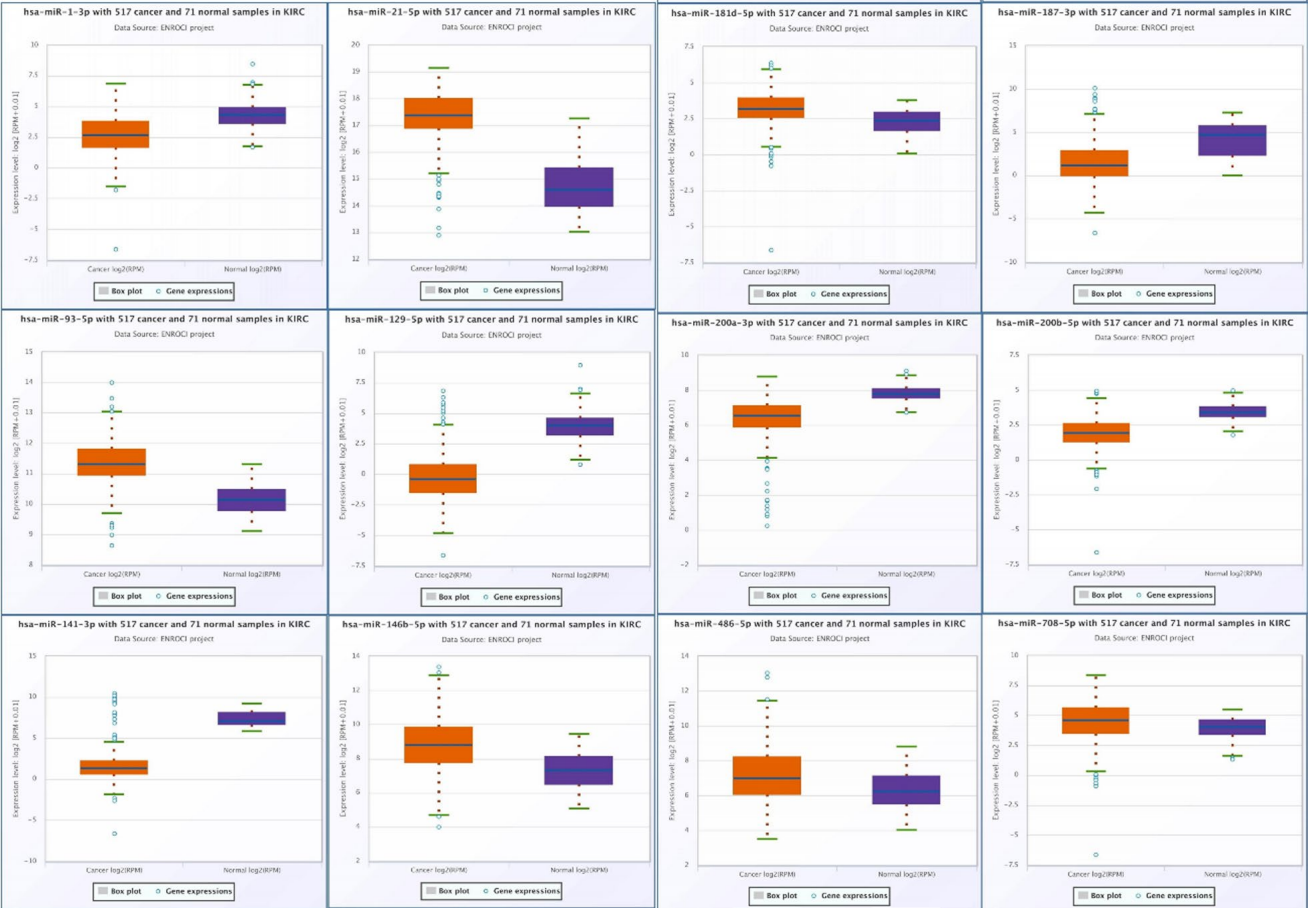
Twelve candidate miRNAs were selected using the ENCORI database, based on the following criteria: p < 0.05 and fold change (FC) of > 1 or <  −1

**Fig. 2:**
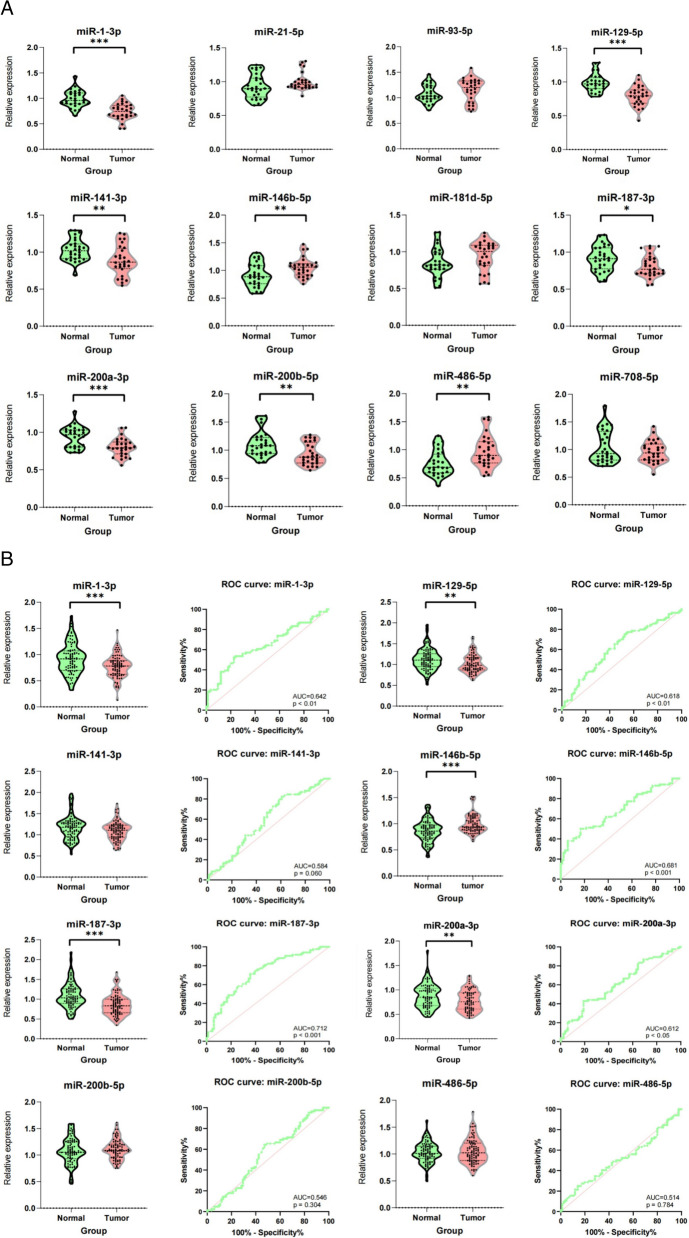
Relative miRNA expression in normal and tumor cell samples. **A** Eight differentially expressed miRNAs (miR-1-3p, miR-129-5p, miR-141-3p, miR-146b-5p, miR-187-3p, miR-200a-3p, miR-200b-5p, and miR-486-5p) were identified in KIRC and NC serum samples (n = 28 each). **B** Five differentially expressed miRNAs (miR-1-3p, miR-129-5p, miR-187-3p, miR-146b-5p, and miR-200a- 3p) were identified in KIRC and NC serum samples (n = 84 each). The p values of ROC curves for miR-1-3p, miR-129-5p, miR-146b-5p, miR-187-3p, and miR-200a-3p were all were < 0.05 and the corresponding area under the curve values were 0.642, 0.618, 0.681, 0.712, and 0.612, respectively.* indicates p < 0.05, ** indicates p < 0.01, ***indicates p < 0.001

KIRC-associated miRNAs were selected from the Gene Expression Omnibus and PubMed databases. Twelve candidate miRNAs were selected for further analysis using the ENCORI database (Fig. 1). In patients with KIRC, six miRNAs (hsa-miR-21-5p, hsa-miR-93-5p, hsa-miR-146b-5p, hsa-miR-181d-5p4, hsa-miR-486-5p, and hsa-miR-708-5p) were upregulated and the other six miRNAs (hsa-miR-1-3p, hsa-miR-129-5p, hsa-miR-141-3p, hsa-miR-187-3p, hsa-miR-200a-3p, and hsa-miR-200b-5p) were downregulated. Using RT-qPCR and the 2 − △△Cq method, we determined the presence of miRNAs in the NC and KIRC samples. We found that eight miRNAs (miR-1-3p, miR-129-5p, miR-141-3p, miR-146b-5p, miR-187-3p, miR-200b-5p, miR-200a-3p and miR-486-5p) were significantly differentially expressed in KIRC (Fig. 2A).

### Diagnostic potential of the candidate miRNAs

We determined the diagnostic potential of the candidate miRNAs. miR-1-3p, miR-129-5p, miR-141-3p, miR-146b-5p, miR-187-3p, miR-200b-5p, miR-200a-3p, and miR-486-5p were differentially expressed in patients with KIRC. Further verification, using RT-qPCR and the 2 − △△Cq method, revealed that miR-1-3p, miR-129-5p, miR-146b-5p, miR-187-3p, and miR-200a-3p were significantly differentially expressed in KIRC. Compared with NCs, in patients with KIRC, miR-146b-5p was upregulated, whereas the other miRNAs were downregulated (Fig. 2B). The AUC values for miR-1-3p, miR-129-5p, miR-146b-5p, miR-187-3p, and miR-200a-3p were 0.642, 0.618, 0.681, 0.712, and 0.612, respectively.

### Combined miRNA panel for the detection of KIRC

**Fig. 3:**
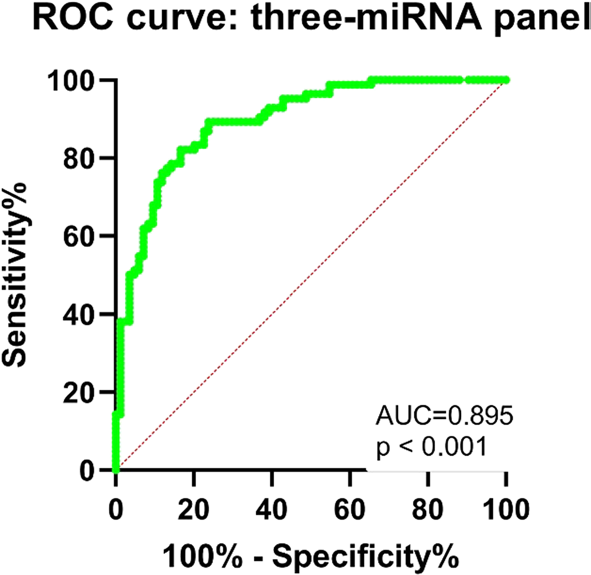
ROC curve evaluation of the three-miRNA panel. This three-miRNA panel contained miR-1-3p, miR-129-5p, and miR-146b-5p, and the AUC for the panel was 0.895 (95% CI: 0.848 to 0.942; sensitivity = 89.30%, specificity = 76.19%). The diagnostic efficacy was evaluated as follows: AUC 0.5–0.7 (low), 0.7–0.9 (medium), and 0.9–1.0 (high)

Considering that miRNA clusters have higher diagnostic accuracy than single miRNA, we evaluated the diagnostic ability of miRNA clusters. With p < 0.01 AUC ranging from 0.848 to 0.942, we found that the miR-1-3p, miR-129-5p, and miR-146b-5p combination may be the most effective biomarker panel for KIRC screening (Fig. 3). The following equation was used to determine the outcome of the final logistic regression model: git (P) = −1.343–3.211 miR-1-3p + 7.623*miR-146b-5p-4.473*miR-129-5p.

### Role of candidate miRNAs

**Fig. 4:**
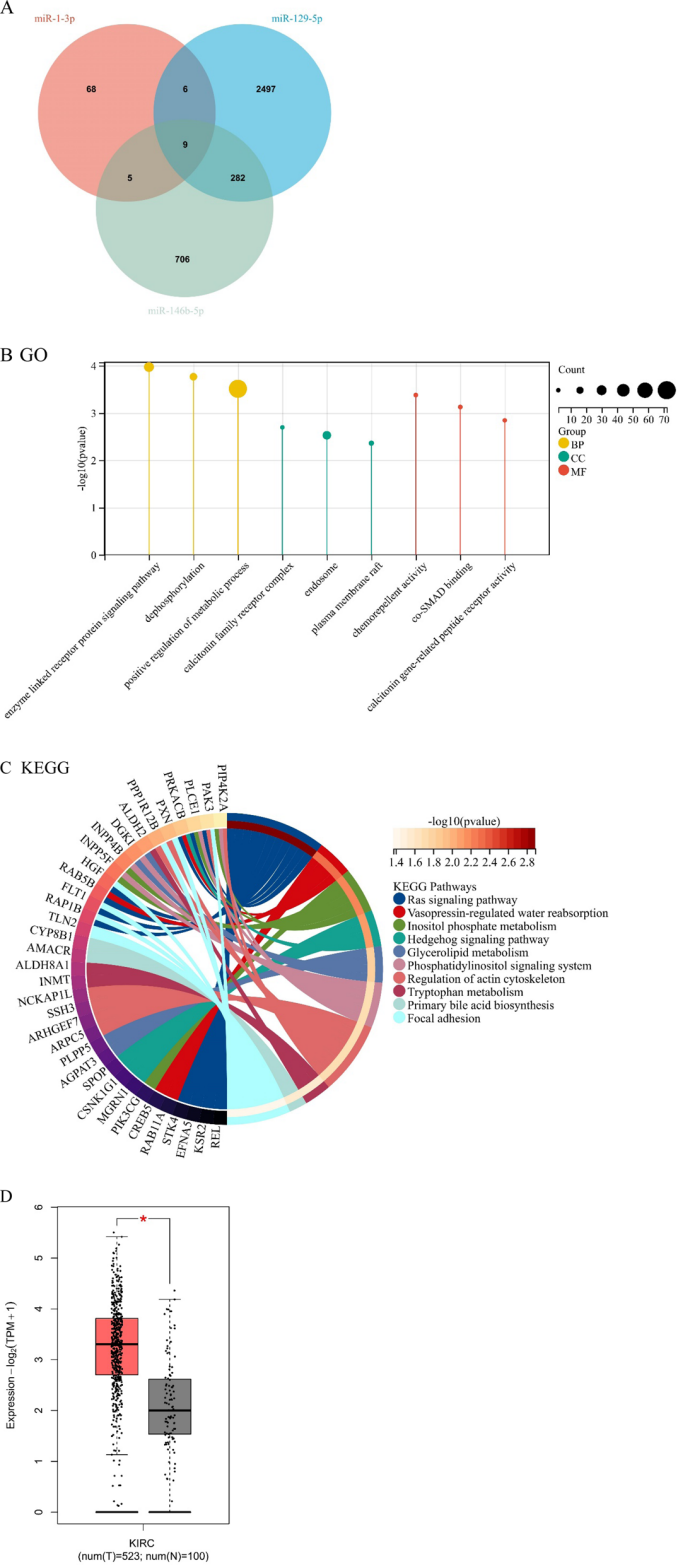
**A** miRNA target genes. We analyzed 302 target genes for GO annotation and KEGG pathway using the Enrichr database. **B** The y-axis represents -log10 (p-value), where a larger -log10 value corresponds to a smaller p-value, indicating a more significant pathway. The x-axis represents the path name. The size of the dots indicated the number of genes. Yellow represents the biological process (BP). Green represents the cellular component (CC). Red represents molecular function (MF). **C** The circle color represents the KEGG path. The color of the left circle from black to light orange represents different genes. The color of the left circle from light red to deep red represents -log10 (p-value). A larger -log10 (p-value) represents a smaller p-value, indicating that the gene is more significant. **D** Nine target genes in all candidate miRNAs (A). Using the GEPIA database, we analyzed the differential expression of nine target genes in KIRC and found that *CREB5* was most significantly related to KIRC based on: match TCGA normal and GTEx data and |log2FC|> 1, p < 0.01. *T* tumor, *N* normal control

Using miRWalk3.0, we predicted the target gene for each candidate miRNA (miR-1-3p, miR-129-5p, and miR-146b-5p). A total of 302 genes were chosen as potential targets because of their involvement in the function of more than two different miRNAs (Fig. 4A). Enrichment analysis was used for GO annotation and KEGG pathway analysis and the function of the target genes was determined (Fig. 4B, C). Nine target genes were successfully identified across all potential miRNAs. We investigated the differential expression of nine target genes in KIRC using the GEPIA database and found that *CREB5* was the most significantly different in KIRC (Fig. 4D, |log2FC|> 1, p < 0.01).

### Survival and prognostic prediction of the candidate miRNAs

**Fig. 5:**
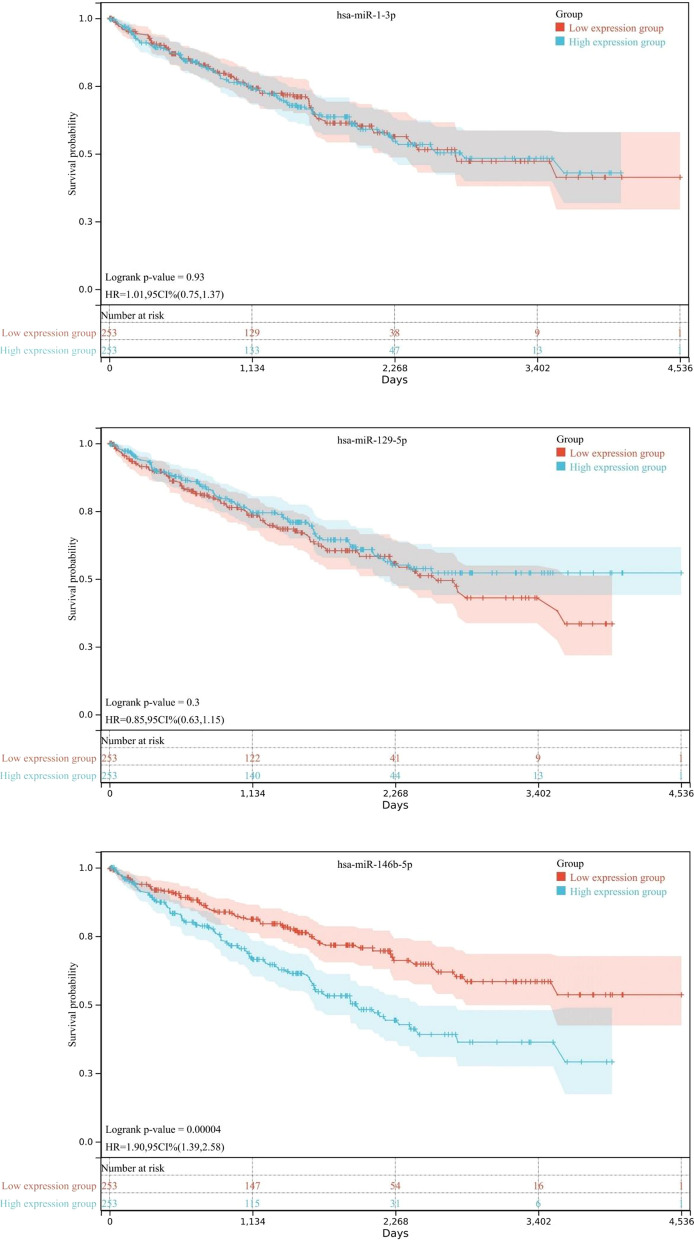
Kaplan–Meier survival curves of three candidate miRNAs. The analysis revealed that miR-146b-5p is significantly associated with KIRC survival rate, and KIRC cases with higher miR-146b-5p expression had a poor prognosis

We analyzed the survival rates of 506 patients with KIRC from the OncoLnc database using Kaplan–Meier survival analysis. Our comparison was based on dichotomized QPCT expression performed using a log-rank test. The results demonstrated that miR-146b-5p was significantly associated with the survival rate of patients with KIRC (log-rank p < 0.01); patients with KIRC and miR-146b-5p overexpression had a poorer prognosis (Fig. 5).

## Discussion

Currently, more than 50% of KIRC cases are detected incidentally through frequent non-invasive radiological examination [4]; therefore, the development of early KIRC detection methods are crucial. miRNAs are highly stable in many biological fluids; thus, they can be used as biomarkers to detect tumor formation [11]. In this study, we aimed to identify KIRC-associated miRNAs. We found that miR-1-3p, miR-129-5p, miR-146b-5p, miR-187-3p, and miR-200a-3p were significantly associated with KIRC development. In addition, we discovered that a biomarker panel consisting of miR-1-3p, miR-129-5p, and miR-146b-5p might be the most effective combination for identifying KIRC. Using Kaplan–Meier survival analysis, we compared the survival rates of 506 patients with KIRC and found that miR-146b-5p was overexpressed in patients with KIRC that had a poor prognosis.

miR-1-3p levels in the serum of patients with KIRC were lower than that in healthy controls, indicating that miR-1-3p may inhibit KIRC development. miR-1-3p has been found to have an inhibitory effect on various malignancies, including hepatocellular carcinoma [19], prostate cancer [20], and bladder cancer [21].

Recently, a long non-coding RNA (lncRNA)-miRNA-mRNA regulatory circuit proposed that miRNAs could be competitively inhibited by lncRNAs as competing endogenous RNAs [22]. Overexpression of LINC00242 in gastric cancer cells inhibits miR-1-3p expression, which in turn adversely regulates G6PD expression. This inhibited both tumor proliferation and aerobic glycolysis [23]. In hepatic carcinogenesis, TUG1 (lncRNA) adsorbs miR-1-3p, promoting IGF1 expression and tumor proliferation [24]. In esophageal squamous cell carcinoma, LINC01518 knockdown upregulated miR-1-3p, which inhibited tumorigenicity [25]. Circular RNAs (circRNA) may exert effects by sponging miRNA, inevitably leading to the derepression of miRNA targets [26]. Glycosyltransferase, C1GALT1, is controlled in bladder cancer (BLCA) by the cHP1BP3 (circRNA produced from HP1BP3) –miR-1-3p axis, which represses and decreases the migratory ability and proliferation of BLCA cells in vitro and in vivo by altering target glycoproteins [27].

Similarly, we found that miR-129-5p was downregulated in KIRC serum than in NCs. Recent research has shown that miR-129-5p has antitumor effects in KIRC [28]. When *SPN* is downregulated, miR-129-5p limits KIRC progression [28].

Docetaxel resistance is promoted in prostate cancer via CAMK2N1 downregulation, which is associated with miR-129-5p, which is in turn associated with tumor drug resistance [29]. miR-129-5p is an essential component of lncRNA-miRNA-mRNA and circRNA-miRNA circuitry regulation. lncARSR acts as a sponge for miR-129-5p, which promotes the metastasis of BLCA and proliferation of BLCA cells. lncARSR sponging miR-129-5p depends on increasing the expression of sex-determining region Y-related high-mobility group box transcription factor 4 (SOX4) [30]. The miR-129-5p that is controlled by circ 0007841 affects the proliferation and metastasis of multiple myeloma [31].

In addition, we found that miR-146b-5p was significantly related to KIRC prognosis.

miR-146b-5p may influence the expression of the *BRCA1* gene to govern the progression of triple negative sporadic breast cancers [32]. Moreover, miR-146b-5p serves as a potential predictive biomarker for triple negative breast cancer relapse [33]. A panel of miRNAs has been used to predict hepatocellular carcinoma [34] and colorectal cancer [35]. miRNAs (miR-1-3p, miR-129-5p, and miR-146b-5p) serve as potential reliable biomarkers for KIRC diagnosis (Fig. 3).

Using the GEPIA database, we found that *CREB5* is a common miR-1-3p, miR-129-5p, and miR-146b-5p target gene. In metastatic castration-resistant prostate cancer, *CREB5* together with *FOXA1* promotes epithelial to mesenchymal transition signaling in AR-positive-resistant cells [36, 37]. *CREB5* directly activates mesenchymal-epithelial transition to promote the invasiveness and metastasis of colorectal cancer [38] and regulates vasculogenic mimicry in breast cancer cells [39]. *CREB5* is a hypoxia-activated transcription factor that is involved in tumorigenesis [39]. The specific loss of the short arm of chromosome 3, which encodes the tumor suppressor gene VHL, is the earliest KIRC tumorigenesis event [40], which encodes the tumor suppressor gene, VHL [41]. Loss of VHL stabilizes the protein levels of the hypoxia­inducible factors, HIF1α and HIF2α, resulting in oxygen loss and tumor cell pseudohypoxia. In pseudohypoxia, HIF1α and HIF2α upregulate the expression of several genes that promote cellular proliferation and angiogenesis, aiding tumorigenesis [42, 43]. *CREB5* has an effect similar to that of VHL; under hypoxic conditions, *CREB5* promotes tumor angiogenesis.

The AUC of a four-miRNA panel for RCC diagnosis containing miR-1-3p, miR-155-5p, miR-200b-3p, and miR-224-5p was 0.903 (95% CI 0.847–0.944; p < 0.001; sensitivity = 75.61%; specificity = 93.67%) [44]. Similarly, the AUC of another four-miRNA panel, containing miR-18a-5p, miR-138-5p, miR-141-3p, and miR-181b-5p, was 0.908 (95% CI 0.852–0.948; sensitivity = 80.77%, specificity = 88.89%) [45]. In our study, the AUC of a three-miRNA panel, including miR-1-3p, miR-129-5p, and miR-146b-5p, for KIRC diagnosis, was 0.895 (95% CI 0.848 to 0.942; sensitivity = 89.30%, specificity = 76.19%). Compared with previous studies, our study focused on KIRC and not on RCC; however, KIRC-associated RNAs are highly correlated with those of RCC. In contrast, miR-1-3p simultaneously emerged in the RCC and KIRC panels; however, other miRNAs did not simultaneously emerge in RCC and KIRC, indicating that miRNAs play different roles in different RCC subtypes. Therefore, the functions of the identified miRNAs in different RCC subtypes should be investigated. In our study, a small study number was used, which is a limitation; therefore, studies involving larger sample numbers are required.

## Conclusions

We found that an miRNA panel, consisting of miR-1-3p, miR-129-5p, and miR-146b-5p, serve as serum biomarkers for the predication of KIRC prognosis. In addition, we found that miR-1-3p serves as a potential biomarker of RCC and KIRC. Finally, we found that *CREB5* serves as a potential target gene for KIRC treatment.

## Data Availability

All relevant data that support the findings of this study are available from the corresponding author upon request.

## Abbreviations

AUC: Area under the ROC curve
BLCA: Bladder cancer
BP: Biological process
CC: Cellular component
CT: Computed tomography
GO: Gene ontology
HDI: Human development index
KEGG: Kyoto encyclopedia of genes and genomes
KIRC: Kidney renal clear cell carcinoma
MF: Molecular function
RCC: Renal cell carcinoma
ROC: Receiver operating characteristic
